# DeepLoc 2.1: multi-label membrane protein type prediction using protein language models

**DOI:** 10.1093/nar/gkae237

**Published:** 2024-04-08

**Authors:** Marius Thrane Ødum, Felix Teufel, Vineet Thumuluri, José Juan Almagro Armenteros, Alexander Rosenberg Johansen, Ole Winther, Henrik Nielsen

**Affiliations:** Section for Bioinformatics, Department of Health Technology, Technical University of Denmark, 2800 Kongens Lyngby, Denmark; Bioinformatics Centre, Department of Biology, University of Copenhagen, 2200 Copenhagen, Denmark; Digital Science & Innovation, Novo Nordisk A/S, 2760 Måløv, Denmark; University of California, San Diego, CA 92093, USA; Bristol Myers Squibb Company, Informatics and Predictive Sciences Research, Calle Isaac Newton 4, Sevilla 41092, Spain; Department of Computer Science, Stanford University, Stanford, CA 94305, USA; Bioinformatics Centre, Department of Biology, University of Copenhagen, 2200 Copenhagen, Denmark; Department of Genomic Medicine, Rigshospitalet (Copenhagen University Hospital), 2100 Copenhagen, Denmark; Section for Cognitive Systems, Department of Applied Mathematics and Computer Science, Technical University of Denmark, 2800 Kongens Lyngby, Denmark; Section for Bioinformatics, Department of Health Technology, Technical University of Denmark, 2800 Kongens Lyngby, Denmark

## Abstract

DeepLoc 2.0 is a popular web server for the prediction of protein subcellular localization and sorting signals. Here, we introduce DeepLoc 2.1, which additionally classifies the input proteins into the membrane protein types *Transmembrane*, *Peripheral*, *Lipid-anchored* and *Soluble*. Leveraging pre-trained transformer-based protein language models, the server utilizes a three-stage architecture for sequence-based, multi-label predictions. Comparative evaluations with other established tools on a test set of 4933 eukaryotic protein sequences, constructed following stringent homology partitioning, demonstrate state-of-the-art performance. Notably, DeepLoc 2.1 outperforms existing models, with the larger ProtT5 model exhibiting a marginal advantage over the ESM-1B model. The web server is available at https://services.healthtech.dtu.dk/services/DeepLoc-2.1.

## Graphical Abstract

**Graphical Abstract:**
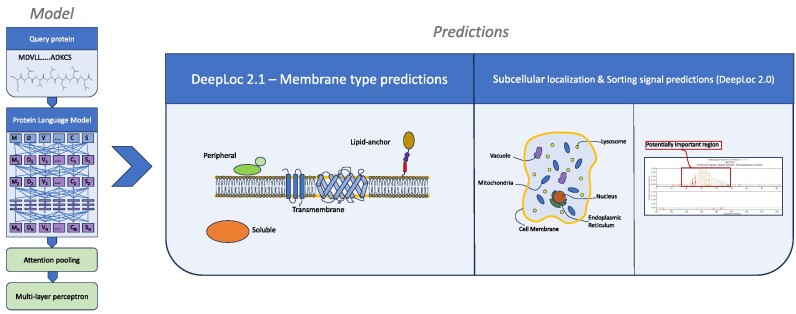

## Introduction

Membrane proteins are essential for a wide range of cellular functions. These functions range from assisting the cell in mediating communication through signaling pathways to facilitating the transportation of macromolecules and maintaining ion gradients across membranes (1). For these reasons, membrane proteins often pose as targets in drug discovery, highlighting their biological importance for proteomics research (2,3).

On a high level, membrane proteins can be categorized into three major classes: peripheral membrane proteins, transmembrane proteins and lipid-anchored proteins (1). This categorization of a protein into one of these classes often provides insight into its functionality and biological properties. Similar to the task of predicting the subcellular localization of eukaryotic proteins, which was the aim of DeepLoc 2.0 (4), having access to tools that can accurately determine the membrane association of a protein in addition to its subcellular localization is highly useful from a medical and biotechnological standpoint.

In DeepLoc 1.0, a binary membrane classification was included in the server’s output, where a protein would be predicted as either membrane-bound or soluble (5), but in DeepLoc 2.0, this functionality was not included. With DeepLoc 2.1, we not only reinstate the capability of predicting membrane proteins, but expand it considerably by also distinguishing between the three types of membrane proteins. Due to the high similarity between these multi-label prediction tasks, the models developed for the prediction of membrane association have been built upon a similar data structure and model architecture. We use sequence-based embeddings from the ProtT5 (6) and ESM-1B protein language models (7) and implement the same model architecture used by DeepLoc 2.0, performing interpretable attention pooling over sequence embeddings with discrete cosine transform regularization.

Various homology and machine learning-based tools exist for the prediction of membrane protein types. The majority of these tools can be categorized as homology-based models, that utilize sequence alignment to search for gene ontology (GO) terms within a database of experimentally annotated proteins to reach a conclusion about the membrane association of a query protein. This property often poses the limitation of homology-based models as high-quality experimental annotations of proteins are often expensive and laborious. Additionally, the databases used for searching GO-terms must be continuously updated to ensure acceptable accuracy of the models as novel proteins and functionality are discovered.

Among the tools that have gained the most attention for the task of predicting membrane protein types are Mem-ADSVM (8), MemPype (9) and MemType-2L (10). Mem-ADSVM is a multi-label homology-based predictor that uses a support vector machine to infer its membrane type predictions exclusively based on the GO-terms of homologous accession numbers from a compact database named ProSeq-GO (8). The MemPype server is a multi-class predictor developed for eukaryotic proteins. The server provides two outputs, i.e. one that is solely based on various machine learning tools and sequence-based inference along with a seperate homology-based output that acts to support the prediction of the ML-based output. The ML-based sequence profiling uses multiple other tools for the prediction of signal peptides, GPI-anchors, and transmembrane domains to arrive at a decision about the membrane type of the query protein (9). MemType-2L is another multi-class predictor that utilizes sequence similarity (10). Instead of searching for homologous sequences using alignment-based methods, it represents known proteins using a pseudo position-specific scoring matrix (Pse-PSSM) and performs *k*-nearest neighbor classification on a Pse-PSSM encoded query protein.

We propose a multi-label prediction tool that relies purely on sequence-based predictions utilizing the advances made in recent years using transformer-based protein language models (11–13). DeepLoc 2.1 builds upon the same template architecture as the previous DeepLoc models for the prediction of subcellular localization. The architecture is defined by three stages. Firstly, a pre-trained protein language model is used to create a feature representation for each amino acid in the sequence (6,7). Secondly, the feature representation is passed to an attention-based pooling stage, which generates a single representation for the entire sequence. Lastly, the attention-based representation is passed to a classifier that predicts the membrane type labels.

## Web server

**Figure 1.**
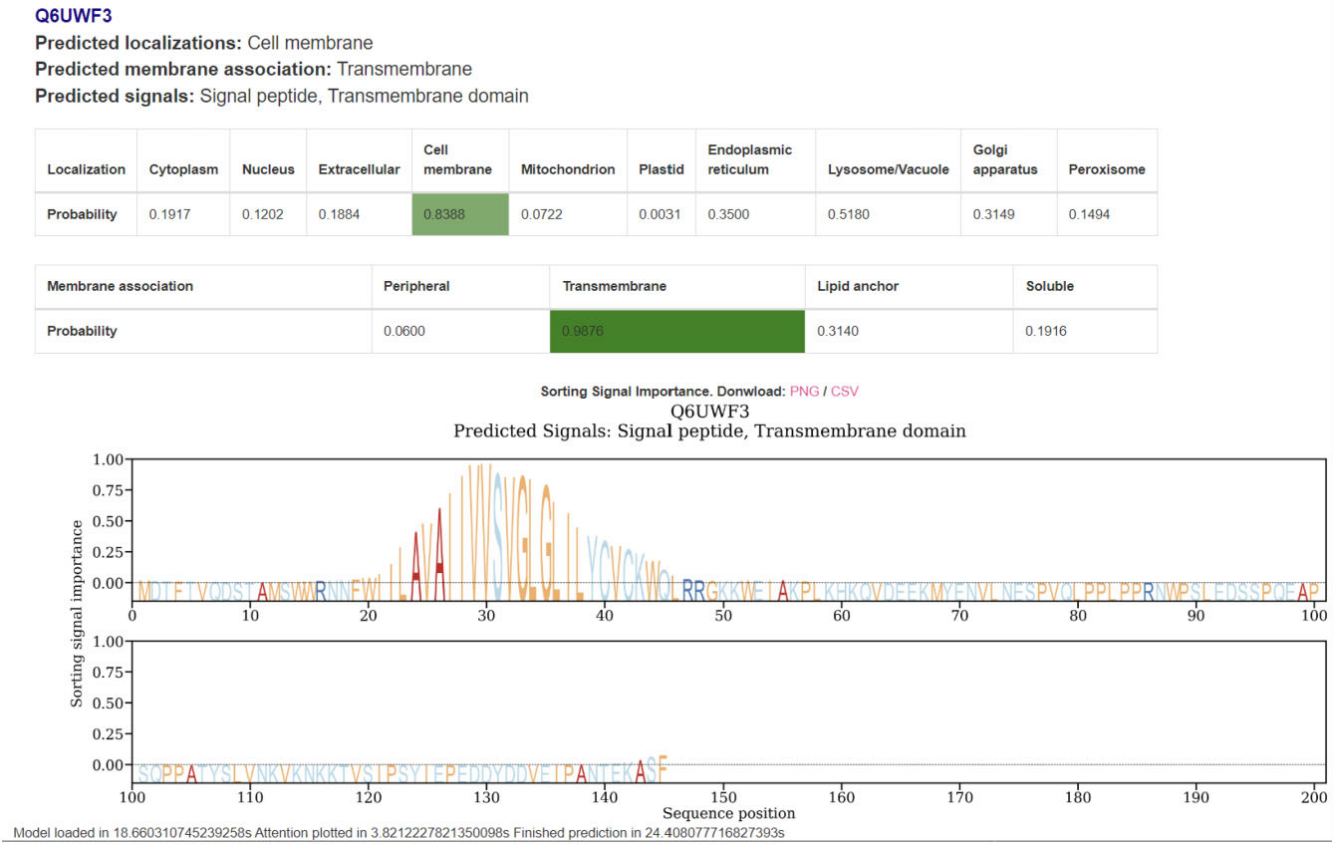
An example display of the predictions from the web server. All results from the tables can be downloaded as a comma-separated file (CSV) at the top of the page, which includes the predictions for the subcellular localization, membrane protein type and sorting signals. The attention plot and attention values can be downloaded separately. The predicted subcellular localization, membrane protein type and sorting signal labels are listed, along with prediction score tables. The predicted locations and membrane protein types in the tables are highlighted in green, with the intensity of the color indicating the certainty of the prediction. If none of the scores surpasses the threshold, the label closest to its threshold is selected. Elevated values in the logo-like plot indicate important regions in the sequence for the subcellular localization prediction, potentially corresponding to sorting signals. This is intended as a general guideline, and for a more in-depth and precise analysis of these signals, specialized tools like SignalP (14), TargetP (15) or NetGPI (16) can be employed.

The web server of DeepLoc 2.1 enables the prediction of the four membrane protein types, *Peripheral membrane protein*, *Transmembrane protein*, *Lipid-anchored protein*, *Soluble protein* (*i.e. non-membrane*), along with the original output of the DeepLoc 2.0 web server that includes prediction of ten subcellular locations and nine sorting signals of a query protein. Additionally, an attention plot of the sequence is provided. Like its predecessor, DeepLoc 2.1 is free and publicly available with no login requirements. Input to the server is provided in FASTA format and the server takes a maximum of 500 sequences per submission. The predicted membrane protein types are displayed in a table highlighted with shades of green for positive labels. The intensity of the green color indicates the certainty of the predicted label to be a true label relative to the threshold value for a positive label for the given membrane protein type, similar to DeepLoc 2.0. DeepLoc 2.1 will always infer at least one positive label for its membrane type prediction, which implies that if the model did not predict any positive label, the inferred membrane protein type will be the one closest to its associated threshold value. Figure 1 showcases an example output of the web server. Estimates of prediction times are provided in Supplementary Table S1.

## Data

We curated a dataset with experimental evidence for membrane protein type labels containing 25 240 eukaryotic protein sequences from the UniProt database (17). All data were extracted from UniProt release 2022_10 and were filtered using the following criteria: Eukaryota (eukaryotes) [2759], not fragments, >40 amino acids and any experimental assertion. From this data the sequences can be categorized into one or multiple of the following four membrane protein types: *Peripheral*, *Transmembrane*, *Lipid-anchor* and *Soluble* (for non-membrane association). Details of the curation process are given in Supplementary Section 1 and Supplementary Tables S2 and S3.

This dataset was split into five partitions using GraphPart (18), with a maximum global sequence identity of 30% between sequences in different partitions, as measured by Needleman–Wunsch alignments. Four of these partitions were used for 4-fold cross-validation, while the last partition including 4933 sequences was used solely for testing (see Table 1). Supplementary Table S4 shows a detailed description of the label distribution of membrane protein types between all partitions.

**Table 1.** The composition of the held-out test set

	*N*
Total	4933
Single-label	4493
Multi-label	440
Peripheral	426
Transmembrane	1516
Lipid anchor	122
Soluble	3312

## DeepLoc 2.1 overview

The DeepLoc 2.1 methodology for predicting the type of membrane protein has three stages that builds upon the pipeline developed for DeepLoc 2.0. The first stage involves creating per-position representations, extracted from a pre-trained transformer-based protein language model for each amino acid in a query protein. During the development of DeepLoc 2.1, four protein language models were assessed and compared for predicting membrane protein types. These included three models of the Evolutionary Scale Modeling project from Meta (7,19), ESM-1B-650M (used for DeepLoc 2.0 and referred to as ESM-1B), ESM-2-650M and the largest model from this project, the ESM-2-15B. Furthermore, we evaluated the performance of the ProtT5-XL-Uniref50, referred to as ProtT5, from the ProtTrans project (6), also used for DeepLoc 2.0. However, only ESM-1B and ProtT5 models are included in the DeepLoc 2.1 web server because no distinct improvements were observed for the membrane protein type predictions with the newer ESM-2 protein language models (19) compared to the previous ESM-1B model. The decision to only include these models was made to maintain compatibility with the existing DeepLoc 2.0 pipeline, which utilizes the ESM-1B and ProtT5 model so that only a single per-position representation for a query protein needs to be computed for the subcellular location and membrane protein type prediction tasks.

The per-position embeddings of a sequence of length *L* undergo attention pooling to compute a single representation vector for the whole sequence. Attention pooling allows the model to focus on regions in the sequence that are important for localization prediction, such as hydrophobic transmembrane stretches or lipid anchor sites. The prediction of attention weights is regularized using a Discrete Cosine Transform (DCT) prior to yield more interpretable attention patterns (20).

Finally, the attention-pooled representation vector is passed to a linear layer that outputs a probability for each label. The loss function used for training the models is a weighted focal loss function (21). Additionally, a probability threshold value is computed for each label independently by optimizing Matthews’ Correlation Coefficient (MCC) on the training data. As opposed to other accuracy metrics, the threshold optimization using the MCC-score better accommodates label imbalance (22).

Details of the DeepLoc 2.1 implementation and training are given in Supplementary Section 2 and Supplementary Table S5, and the model architecture is illustrated in Supplementary Figure S1.

## Results and discussion

The two models developed for DeepLoc 2.1, employing the ESM-1B (high-throughput) and the ProtT5 (high-quality) protein language models demonstrate nearly identical performance across the assessed metrics seen in Table 2 when evaluated on the test set (Table 1). The larger model, ProtT5, appears to have a marginal edge over the ESM-1B across the majority of the performance metrics. Considering the MCC for the different labels it can be seen that ProtT5 is slightly less prone to inferring wrong predictions for the Peripheral and Soluble labels. These two classes exhibit a significant biochemical overlap, posing a challenge for the models to effectively differentiate between them, and for this purpose the larger model seems to possess a marginal advantage.

**Table 2.** Performance metrics of the DeepLoc 2.1 models, ESM-1B and ProtT5

	Count	ESM-1B	ProtT5
Accuracy	4933	0.87	**0.88**
Subset acc.	4933	0.79	**0.80**
Jaccard	4933	0.83	**0.84**
F1_*micro*_	4933	0.88	**0.89**
F1_*macro*_	4933	0.74	**0.75**
Predicted/true	5280	**1.06**	**1.06**
**MCC per location**			
Peripheral	426	0.37	**0.39**
Transmembrane	1516	**0.95**	0.94
Lipid anchor	122	0.63	**0.66**
Soluble	3312	0.80	**0.82**
**Specificity per location**			
Peripheral	426	**0.89**	**0.89**
Transmembrane	1516	**0.99**	0.98
Lipid anchor	122	0.98	**0.99**
Soluble	3312	0.88	**0.89**
**Sensitivity per location**			
Peripheral	426	0.58	**0.61**
Transmembrane	1516	**0.96**	**0.96**
Lipid anchor	122	**0.75**	0.66
Soluble	3312	**0.93**	**0.93**
**Precision per location**			
Peripheral	426	0.33	**0.35**
Transmembrane	1516	**0.97**	0.95
Lipid anchor	122	0.55	**0.67**
Soluble	3312	**0.94**	**0.94**

To establish a baseline, the models were evaluated against an alignment-based baseline, utilizing MMSeqs2 (23) for sequence alignment. Positive labels for each specific sample were deduced based on the true labels of the sequence with the highest alignment score in any other partition. A comparison between the findings in Table 2 and the baseline results, described in Supplementary Section 3 and Supplementary Table S6, reveals that the models significantly outperform these baseline predictions, indicating that simple homology-based models lack the contextual depth necessary for accurate predictions.

To evaluate the performance of DeepLoc 2.1 we have compared our model to the Mem-ADSVM, MemPype and MemType-2L methods, which are all established servers for the task of predicting the membrane association of a protein. As mentioned, all of these servers are homology-based to a certain extent, which has led us to construct different sub-sets of our test set in order to achieve a fair comparison, with as little overlap as possible between the test set and the database used by the various models. More details on this can be found in Supplementary Section 4 and Supplementary Tables S7, S8 and S9. Additionally, some of the models that have been included for comparison have different output characteristics. Both MemPype and Memtype-2L are single-label multi-class predictors, which implies that we have excluded all multi-label samples for comparison with these models. To retrieve single-label predictions for our models, we applied the softmax activation function to the raw outputs of the models that were trained for multi-label prediction.

### Model comparison

As seen in Table 3, all performance metrics indicate a considerable advantage in favor of the protein language models employed by DeepLoc 2.1. The only metric where Mem-ADSVM demonstrates a performance approaching that of DeepLoc 2.1 is in terms of subset accuracy. The reason for this lies in the decision making mechanism of the Mem-ADSVM server, where if a protein has been predicted as soluble the model will not proceed to make any further predictions. Due to the biochemical overlap between the soluble and peripheral class coupled with the significant sample imbalance, where soluble proteins are eight times more abundant than peripheral proteins in the dataset, a decision mechanism, such as that employed by Mem-ADSVM, could potentially exhibit a bias towards the majority class. However, this characteristic of Mem-ADSVM contradicts its assertion of being a multi-label predictor, particularly in light of the fact that a majority of the multi-label samples emerge from the soluble and peripheral classes (refer to Supplementary Section 1 for further details on the dataset and label distribution).

**Table 3.** Comparing performance metrics of the Mem-ADSVM server, a multi-label homology-based predictor for all four types of membrane associativity, to the MCC threshold optimized models of DeepLoc 2.1. The test set contains 803 samples from partition V that were included in the UniProtKB database after 2014

	Count	Mem-ADSVM	ESM-1B	ProtT5
Accuracy	803	0.80	0.89	**0.91**
Subset acc.	803	0.77	0.83	**0.85**
Jaccard	803	0.69	**0.86**	**0.86**
F1_*micro*_	803	0.81	0.90	**0.91**
F1_*macro*_	803	0.61	0.75	**0.76**
**MCC per location**				
Peripheral	46	0.23	0.31	**0.37**
Transmembrane	228	0.69	**0.92**	**0.92**
Lipid-anchor	17	0.50	**0.76**	0.74
Soluble	558	0.59	**0.83**	**0.83**

Comparing our models to the MemPype server also shows a considerable advantage for DeepLoc 2.1 across all reported metrics (see Table 4). As the MemPype server is a single-label predictor and additionally does not distinguish between soluble and peripheral membrane proteins, we decided to construct a merged class for samples that had true positive labels for the peripheral and/or soluble class and recorded a correct prediction of the merged class if one of the two classes was correctly predicted.

**Table 4.** Comparing performance metrics of the MemPype server, a single-label membrane-type predictor for eukaryotic proteins with three types of membrane associativity, to the MCC threshold optimized models of DeepLoc 2.1. The test set contains 4431 samples from partition V, constituted by eukaryotic single-label samples, and multi-label samples positive for the peripheral and soluble class (see Supplementary Section 4 for construction of the test set)

	Count	MemPype	ESM-1B	ProtT5
Accuracy	4431	0.87	**0.97**	**0.97**
Jaccard	4431	0.79	**0.95**	**0.95**
F1_*macro*_	4431	0.72	0.83	**0.85**
**MCC per location**				
Peripheral/soluble	3042	0.75	**0.94**	**0.94**
Transmembrane	1369	0.76	**0.95**	**0.95**
Lipid-anchor	56	0.43	0.54	**0.59**

A comparison of the DeepLoc 2.1 models against the MemType-2L model shows superior performance of the models based on the transformer protein language models for every metric recorded (see Table 5). Relatively low MCC scores are observed for the single-label case of peripheral membrane proteins, and here the performance of the MemType-2L model approaches that of the DeepLoc 2.1 models. This can be attributed to the fact that the DeepLoc 2.1 models appear to be biased towards the soluble class when constrained to make only single-label predictions. This assertion is supported by the data presented in Table 2, where the sensitivity score for the peripheral class of proteins is notably low. According to the definition of sensitivity, this suggests a significant number of false negative labels are predicted for the peripheral class. Conversely, Table 2 indicates a rare occurrence of false positives for the peripheral class, as evidenced by the specificity for this class being comparable to that of the soluble class for the DeepLoc 2.1 models.

**Table 5.** Comparing performance metrics of the MemType-2L server, a single-label predictor for all four types of membrane associativity, to the MCC threshold optimized models of DeepLoc 2.1. The test set contains 4414 single-label sequences with a length above 50 amino acids

	Count	MemType-2L	ESM-1B	ProtT5
Accuracy	4414	0.78	0.92	**0.93**
Jaccard	4414	0.67	0.87	**0.88**
F1_*macro*_	4414	0.53	0.71	**0.72**
**MCC per location**				
Peripheral	183	0.22	0.26	**0.30**
Transmembrane	1337	0.69	**0.95**	**0.95**
Lipid-anchor	53	0.22	**0.63**	**0.63**
Soluble	2841	0.60	0.86	**0.87**

## Conclusion

In addition to the already existing DeepLoc 2.0, used for predicting the subcellular localization of eukaryotic proteins, we include state-of-the-art prediction of the membrane association of a query protein. The models developed for prediction of membrane-association build upon the same methodology as DeepLoc 2.0, utilizing transformer protein language models, and we show that these purely sequence-based models outperform every other available tool for membrane protein type predictions.

## Supplementary Material

gkae237_Supplemental_File

## Data Availability

The data used for training and testing are available at https://services.healthtech.dtu.dk/services/DeepLoc-2.1/.
