# Supplementary material for: DeepLoc 2.1: multi-label membrane protein type prediction using protein language models
Source: Nucleic Acids Res. 2024 Apr 8;52(W1):W215–20. doi: 10.1093/nar/gkae237 (PMC11223819; doi:10.1093/nar/gkae237)
Supplement: gkae237_Supplemental_File [file gkae237_supplemental_file.pdf]

SUPPLEMENTARY MATERIAL FOR DEEPLOC 2.1

	ESM1b	ProtT5
Short sequences (Average length: 100)		
Model load time (s)	22.72	41.85
Prediction time (s / seq)	4.43	3.93
Plot time (s / seq)	2.56	6.06
Long sequences (Average length: 399)		
Model load time (s)	16.34	45.09
Prediction time (s / seq)	11.48	13.71
Plot time (s / seq)	8.50	8.35

Supplementary Table 1. Estimated time usage on the web server per sequence. The time for prediction and plotting increases proportionally with the number of sequences, while the model load time is constant for any number of sequences.

1 DATA CURATION AND PARTITIONING

To obtain a high-quality dataset we performed various steps to curate the data and ended with a final dataset containing 25,240 eukaryotic protein samples (see Supplementary Table 2). From the raw dataset, we first removed duplicates arising from overlaps between the search criteria at UniProtKB. From the unique samples, we meticulously removed positive labels where the subcellular location was pointing to isoforms (as only canonical sequences were retained in the dataset). Furthermore, all multi-label (>1 positive label) samples were screened and excluded if they exhibited any of the following terms in their annotation for subcellular localization: isoform, cleav[ed, ing], secreted form, process[ed] and shed[ded, ding] (stemmed words used for screening). The result from this screening led to the deletion of 349 positive labels and a total loss of 16 samples. Five partitions were constructed following homology partitioning using GraphPart (1). A maximum pairwise cross-partition identity of 30% was employed which led to the removal of 387 samples.

At the time of homology partitioning we were not aware of the problem arising with multi-chain samples. As it proved laborious to apply proper heuristics to cut these proteins while retaining each chain's true membrane association, we removed these samples from the partitions to avoid introducing any artificial multi-label samples.

Moreover, sequences containing ambiguous amino acids [B, U, Z, X] were also removed after homology partitioning.

Lastly, prokaryotic samples were also removed. Prokaryotic samples were initially included for other experiments during training. However, DeepLoc 2.1 was solely trained and based on eukaryotic data.

Data curation	Count	Comment
Retrieved samples	32,235	Raw dataset from UniProtKB. Containing duplicates due to overlap between search criteria
Unique samples	28,433	Removal of duplicates
Isoform and other removal	28,417	Removing positive labels where subcellular location points to isoform or other causes
Homology partitioning	28,030	Homology partitioning using GraphPart. 5 partitions with a maximum pairwise cross-partition identity of 30%
Multi-chain removal	27,525	Removal of multi-chain samples
Ambiguous removal	27,461	Removal of samples containing ambiguous AAs (B, U, Z, X)
Eukaryotic samples	25,240	Removal of prokaryotic samples

Supplementary Table 2. Steps of data curation.

2 DEEPLOC 2.1: IMPLEMENTATION DETAILS

2.1 Transformer models

We use two publicly available transformer models, the 33-layer ESM-1B model with 650M parameters (2), and the 3B parameter ProtT5-XL-UniRef50 model (3), referred to as ESM-1B and ProtT5.

As the ESM-1B model is not able to generate sequence embeddings for sequences longer than 1022 residues, we decided to cut longer sequences into shorter segments and merge them together afterwards, to retain long sequences for the training of this model. To avoid getting cuts near the ends of the sequences, which could potentially lead to artifacts in the sequence domains expressing potential sorting or signal sequences, we employed a method to ensure sequences were cut closer to the middle of the protein.

2.2 Implementation details

For details regarding the implementation and theory behind the focal loss function and DCT-prior-based regularization, that has been employed in the architecture of DeepLoc 2.1, please refer to the Supplementary Material of DeepLoc 2.0 (4), as the properties of the implementation used in DeepLoc 2.1 are identical to those used in DeepLoc 2.0.

2.3 Training details

Every model was trained for a maximum of 30 epochs if training had not been ceased by an early stopping criterion implemented with a patience of 5 epochs. The focal loss (see Section 2.2) was used for the AdamW optimizer. We implemented a learning rate scheduler for the optimizer, which would halve the learning rate if no improvement in the focal loss was observed for four epochs. The training was performed using 4-fold cross-validation, leaving out the fifth partition solely for testing and benchmarking against external tools. The PyTorch-lightning (5) library was used for the training and testing of the models. The training was carried out on an HPC-cluster using Nvidia Tesla V100 GPUs of 16GB VRAM.

2.4 Model architecture and hyperparameter optimization

The models developed for DeepLoc 2.1 generally follow the same methodology and architecture as the models of

Location	No. of proteins	Sublocations
Peripheral	2189	Peripheral membrane protein [SL-9903]
Lipid-anchor	792	Lipid-anchor [SL-9901]
Transmembrane	7106	Multi-pass membrane protein [SL-9909], Single-pass membrane protein [SL-9904]
Soluble	17377	Mitochondrion matrix [SL-0170], Plastid stroma [SL-0215], Plastid thylakoid lumen [SL-0309], Cytoplasm [SL-0086], Nucleolus [SL-0188], Nucleoplasm [SL-0190], Secreted [SL-0243], Endoplasmic reticulum lumen [SL-0096], Lysosome lumen [SL-0156], Golgi apparatus lumen [SL-0133], Peroxisome matrix [SL-0202], Periplasm [SL-0200]

Supplementary Table 3. UniProt dataset: Number of proteins and translation between membrane protein type and UniProt sublocations.

	N	μ	I	II	III	IV	V
Eukaryota	25240	5048	4735	5162	4775	5635	4933
Single-label	23026	4605	4288	4758	4373	5114	4493
Multi-label	2214	443	447	404	402	521	440
Peripheral	2189	438	437	415	419	492	426
Transmembrane	7106	1421	1374	1449	1390	1377	1516
Lipid-anchor	792	158	165	130	102	273	122
Soluble	17377	3475	3208	3574	3266	4017	3312

Supplementary Table 4. Distribution of data between partitions. Partition **V** is the held-out test set, while the other partitions were used for cross-validation during training. μ is the average.

DeepLoc 2.0. A schematic overview of the models can be seen in Figure 1 in the Appendix Section 5.1. For hyperparameter optimization we made use of the Optuna framework (6) in combination with PyTorch-Lightning. Optuna employs a Tree-Structured Parzen Estimator (TPE) algorithm for sampling new hyperparameters between each trial, to efficiently narrow down the search space for optimal hyperparameters. The hyperparameters that we decided to include can be seen in Table 5 along with their search space.

Hyperparameter	Range	Step	Distribution
Batch size	[128 : 256]	16	Discrete uniform
Hidden layers	[0 : 2]	1	Discrete uniform
Hidden size	[48 : 512]	16	Discrete uniform
Dropout	[0.0 : 1.0]	-	Continuous uniform
Learning rate	$[10^{-4} : 10^{-2}]$	-	Reciprocal
Attention size	[128 : 512]	16	Discrete uniform
Regularization factor	[0.05 : 0.5]	-	Continuous uniform

Supplementary Table 5. Overview of hyperparameters that were tuned during training along with their search space.

A pruner was also implemented in the objective function for the hyperparameter optimization task, to interrupt unpromising trials caused by a choice of less optimal hyperparameters during hyperparameter optimization, to reduce training time (7). Moreover, dropout was applied to the outputs of the attention head and other hidden layers between the attention head and the final output layer to improve generalization and reduce the risk of overfitting.

	MCC	Spe	Sen	PPV
Peripheral	0.15	0.93	0.29	0.13
Transmembrane	0.29	0.76	0.58	0.36
Lipid anchor	0.05	0.97	0.10	0.06
Soluble	0.24	0.55	0.73	0.86

Supplementary Table 6. kNN baseline (k=1) results using MMSeqs2 for sequence alignment.

3 BASELINE EVALUATION

Baseline assessment was carried out using MMSeqs2 (8) for sequence alignment along with a alignment-based classifier, where positive labels were inferred where the sequence from another partition with the highest alignment score had true positive labels. The results from this analysis can be seen in Supplementary Table 6 and be compared to Table 2 in the main article.

4 BENCHMARKING EXISTING METHODS

To benchmark DeepLoc 2.1 against other established tools for membrane protein type predictions we had to modify the test set (partition V in Table 4), in order to allow a fair comparison. This was done to avoid reporting artificially inflated performance of the homology-based models caused by “homology-leakage” between the test set and the search-database used by the model. MemPype (9) also has a different structured output format, as it does not distinguish peripheral membrane proteins from soluble proteins. Additionally, the MemPype and Memtype-2L (10) servers are single-label predictors, which the comparison also had to account for.

For external model comparison, we used the Selenium library in Python to automate submitting sequences to servers and extracting predictions.

4.1 Mem-ADSVM

Mem-ADSVM is a two-layer multi-label homology-based model that uses a support vector machine (SVM) to make its membrane-type prediction (11). It does so based on frequencies of occurrences of the associated GO-terms of the sequence. The GO-terms are retrieved from a compact database, referred to as the ProSeq-GO database, by searching for homologous sequences. We did not have access to the database that Wan et al. are referring to regarding this model (11), and therefore we could not assess which samples from the independent test set that were possibly present in the database, giving the search engine a huge advantage when comparing performance. Furthermore, at the time of submitting this paper we are no longer able to access the server and it does not appear to be available anymore. Evaluating the models on the entire test set showed that the Mem-ADSVM was outperforming our models. To assess if the high performance was due to homology overlap between the test set and ProSeq-GO database, we removed all samples from the test set that were included in the database of UniProt before 2015 (assuming the database had not been updated since the release of the paper). This left us with a subset of 803 independent samples. The new comparison led to highly different results, yielding a more truthful measure of the generalization capability of Mem-ADSVM. The output of the Mem-ADSVM server is reported in a format that is conveniently translated into the four membrane protein types that DeepLoc 2.1 distinguishes between. The translation between outputs used for the comparison can be seen in Table 7.

4.2 MemPype

The MemPype server is a single-label predictor developed for membrane-type predictions of eukaryotic proteins (9). The model uses two pipelines for its predictions. The main pipeline, that we compare our performance to, includes a multi-step prediction stage, that utilizes other available prediction tools for various tasks, e.g. signal peptide prediction with the SPEPlip server (12) and prediction of GPI-anchor propeptide with PredGPI (13). For comparison we use the specific output under the prediction summary of the server. The output distinguishes different types of transmembrane and lipid-anchored proteins, but the outputs are straightforwardly converted to the classes used for this project. The translation between the MemPype and DeepLoc outputs can be seen in Table 8. As the server is designed specifically for single-label predictions and accommodates only eukaryotic sequences, we isolated eukaryotic accessions from the test set featuring single-label annotations for peripheral, transmembrane, and lipid-anchored proteins. In addition to these server specifications, the MemPype pipeline lacks the ability to differentiate between non-membrane proteins and peripheral membrane proteins. Consequently, the server consistently attempts to infer some membrane type for the protein. Considering a protein that is exclusively positive for the soluble class the MemPype model will generate an output that incorporates phrases such as “cell membrane”, “internal membrane”, or “organelle membrane”, and concludes by adding the term “globular”. Due to this limitation, we decided to also include multi-label samples positive for peripheral and soluble labels, and merge these with the single-label peripheral and soluble classes, resulting in a multi-class classification encompassing three membrane types for comparative analysis. This approach yielded a test set comprising 4431 samples. As a consequence of this class-construction, we merged the multi-label Sigmoid predictions of samples with a true label for the soluble and peripheral classes, and inferred a correct prediction if our models had predicted one of the two positive classes.

4.3 MemType-2L

MemType-2L is a two-layer single-label predictor that employs a pseudo position-specific scoring matrix (Pse-PSSM) and optimized evidence-theoretic K nearest neighbors ensemble classifier (OET-KNN) (10). Similarly to the other tools that were assessed for comparison, this model also distinguishes between different types of transmembrane and lipid-anchored membrane proteins that are conveniently translated into the membrane classes of this project. The translation between the MemType-2L and DeepLoc outputs can be seen in Table 9. To allow a fair comparison we removed all multi-label samples from the independent test set along with sequences shorter than 50 AAs which was the shortest sequence length allowed for submission on the server. This resulted in a test set of 4414 sequences. Finally, we applied SoftMax to the raw outputs of the final layer of DeepLoc 2.1 to get multi-class predictions.

4.4 Non-available models

In the literature various other models were also described, and stated to be, or become, public available. However, most of these models are currently inaccessible, due to expired or non-working server-links. Additionally, some models were also not relevant for comparison, as they infer only membrane bound or non-membrane bound. In situations where servers did not appear available or the links seemed to not be working, we made attempts to contact the authors for access to the model. Despite our efforts we did not receive any responses, and therefore we excluded those models from our analysis. The models we tried to access were;

- iMem-Seq (14) (link not working),
- iMem-2SLAAC (15) (establishment of web server mentioned as a future work),
- PMMBF (16) (establishment of web server mentioned as a future work),
- BinMemPredict (17) (link not working),
- ProtLoc (18) (from 1997, deemed outdated from the results of other studies (15)),
- Toot-M (19) (not included as it only infers membrane-bound or not),
- Ali and Hayat (20) (establishment of web server mentioned as future work), and

DeepLoc Location	Mem-ADSVM Location
Peripheral	Peripheral
Lipid-anchor	Lipid-anchor, GPI-anchor
Transmembrane	Single-pass type I, Single-pass type II, Single-pass type III, Single-pass type IV, Multi-pass
Soluble	Soluble (globular)

Supplementary Table 7. DeepLoc to Mem-ADSVM mapping.

DeepLoc Location	MemPype Location
Peripheral/Soluble	Globular
Lipid-anchor	GPI-anchor
Transmembrane	Transmembrane

Supplementary Table 8. DeepLoc to MemPype mapping.

- Han et al. (21) (link not working).

4.5 Performance metrics

We use the following metrics to comprehensively quantify the classification performance on the datasets:

- Number of predicted labels: Averaged over all predictions, this demonstrates the bias of the predictor.
- Accuracy: Requires the exact location(s) to be predicted. Since the dataset is skewed towards proteins with single localization, this metric provides an advantage to single-label predictors.
- Subset accuracy: Determined by calculating the number of predictions in which all labels for each instance were accurately predicted and then dividing that by the total number of instances. This metric is particularly employed in the assessment of multi-label predictions, as opposed to multi-class predictions where each instance always has only one positive label.
- Jaccard: Overlap between the actual and predicted labels over their union.
- MicroF1: F1 score considering the total number of true positives, false negatives, and false positives.
- MacroF1: F1 score computed for each class and then averaged, providing equal emphasis on rare and frequent classes.
- Matthews Correlation Coefficient: Measured for each class, it requires the model to perform well on all four confusion matrix entries.
- Specificity, Sensitivity and Precision: Measured for each class independently.

To fine-tune the performance we optimized the prediction output threshold for the models by maximizing the MCC scores on partitions I-IV. The prediction thresholds are shown on the output page after submitting proteins to the web server.

DeepLoc Location	MemType-2L Location
Peripheral	Peripheral
Lipid-anchor	Lipid-anchor, GPI-anchor
Transmembrane	Single-pass type I, Single-pass type II, Single-pass type III, Single-pass type IV, Multi-pass
Soluble	Non-membrane

Supplementary Table 9. DeepLoc to MemType-2L mapping.

5 APPENDIX

5.1 Model architecture

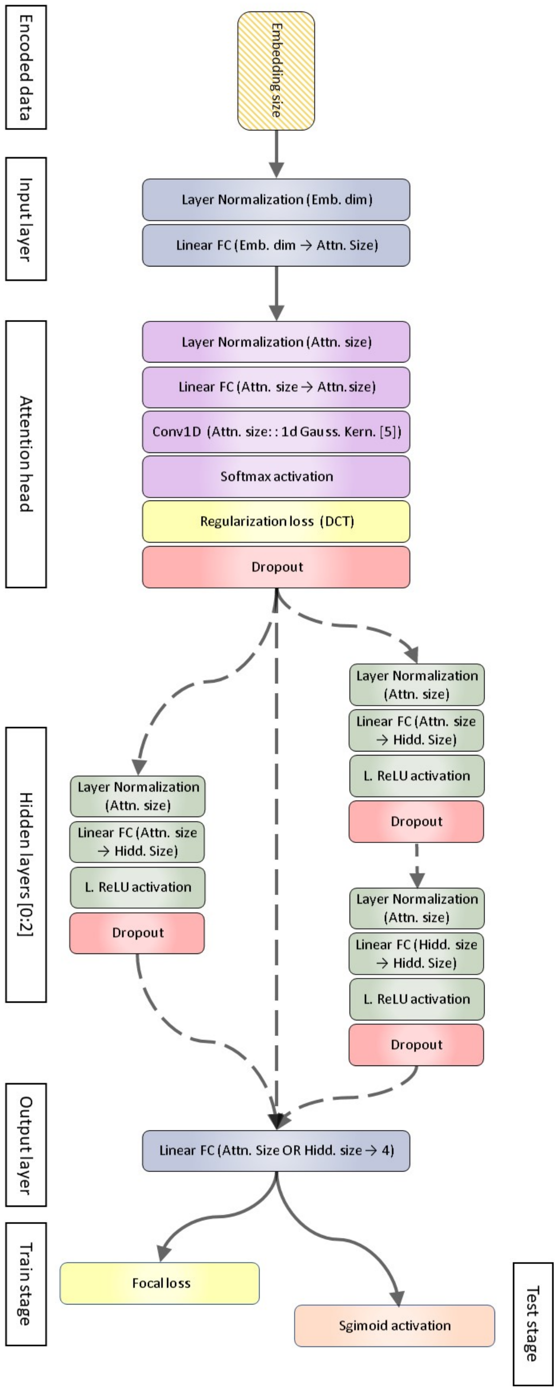

Supplementary Figure 1. General model architecture. Dimensions of layers are not noted in the figure due to variable sizes of embedding inputs and hyperparameter tuning of numbers and sizes of hidden layers.

REFERENCES

1. Teufel, F., Gíslason, M. H., Almagro Armenteros, J. J., Johansen, A. R., Winther, O., and Nielsen, H. (2023) GraphPart: homology partitioning for biological sequence analysis. *NAR genomics and bioinformatics,* **5**(4), lqad088 [PubMed:37850036] [PubMed Central:PMC10578201] [doi:10.1093/nargab/lqad088].
2. Rao, R., Meier, J., Sercu, T., Ovchinnikov, S., and Rives, A. (2020) Transformer protein language models are unsupervised structure learners. *bioRxiv,* p. 2020.12.15.422761 [doi:10.1101/2020.12.15.422761].
3. Elnaggar, A., Heinzinger, M., Dallago, C., Rehawi, G., Yu, W., Jones, L., Gibbs, T., Feher, T., Angerer, C., Steinegger, M., Bhowmik, D., and Rost, B. (2022) ProtTrans: Toward Understanding the Language of Life Through Self-Supervised Learning. *IEEE Transactions on Pattern Analysis and Machine Intelligence,* **44**(10), 7112–7127 [PubMed:34232869] [doi:10.1109/TPAMI.2021.3095381].
4. Thumuluri, V., Almagro Armenteros, J. J., Johansen, A. R., Nielsen, H., and Winther, O. (2022) DeepLoc 2.0: multi-label subcellular localization prediction using protein language models. *Nucleic Acids Research,* **50**(W1), W228–W234 [PubMed:35489069] [PubMed Central:PMC9252801] [doi:10.1093/nar/gkac278].
5. Falcon, W. and The PyTorch Lightning team (2019) PyTorch Lightning. [doi:10.5281/zenodo.3828935].
6. Akiba, T., Sano, S., Yanase, T., Ohta, T., and Koyama, M. (2019) Optuna: A next-generation hyperparameter optimization framework. In *Proceedings of the 25th ACM SIGKDD international conference on knowledge discovery & data mining* [doi:10.1145/3292500.3330701] pp. 2623–2631.
7. Li, L., Jamieson, K., Rostamizadeh, A., Gonina, E., Ben-Tzur, J., Hardt, M., Recht, B., and Talwalkar, A. (2020) A system for massively parallel hyperparameter tuning. In Dhillon, I., Papailiopoulos, D., and Sze, V., (eds.), *Proceedings of Machine Learning and Systems*, Vol. 2, pp. 230–246.
8. Steinegger, M. and Söding, J. (2017) MMseqs2 enables sensitive protein sequence searching for the analysis of massive data sets. *Nature biotechnology,* **35**(11), 1026–1028 [PubMed:29035372] [doi:10.1038/nbt.3988].
9. Pierleoni, A., Indio, V., Savojardo, C., Fariselli, P., Martelli, P. L., and Casadio, R. (2011) MemPype: a pipeline for the annotation of eukaryotic membrane proteins. *Nucleic acids research,* **39**(suppl_2), W375–W380 [PubMed:21543452] [PubMed Central:PMC3125734] [doi:10.1093/nar/gkr282].
10. Chou, K.-C. and Shen, H.-B. (2007) MemType-2L: a web server for predicting membrane proteins and their types by incorporating evolution information through Pse-PSSM. *Biochemical and biophysical research communications,* **360**(2), 339–345 [PubMed:17586467] [doi:10.1016/j.bbrc.2007.06.027].
11. Wan, S., Mak, M.-W., and Kung, S.-Y. (2016) Mem-ADSVM: A two-layer multi-label predictor for identifying multi-functional types of membrane proteins. *Journal of theoretical biology,* **398**, 32–42 [PubMed:27000774] [doi:10.1016/j.jtbi.2016.03.013].
12. Fariselli, P., Finocchiaro, G., and Casadio, R. (2003) SPEPlip: the detection of signal peptide and lipoprotein cleavage sites. *Bioinformatics,* **19**(18), 2498–2499 [PubMed:14668245] [doi:10.1093/bioinformatics/btg360].
13. Pierleoni, A., Martelli, P. L., and Casadio, R. (2008) PredGPI: a GPI-anchor predictor. *BMC bioinformatics,* **9**(1), 1–11 [PubMed:18811934] [PubMed Central:PMC2571997] [doi:10.1186/1471-2105-9-392].
14. Xiao, X., Zou, H.-L., and Lin, W.-Z. (2015) iMem-Seq: a multi-label learning classifier for predicting membrane proteins types. *The Journal of membrane biology,* **248**, 745–752 [PubMed:25796484] [doi:10.1007/s00232-015-9787-8].
15. Arif, M., Hayat, M., and Jan, Z. (2018) iMem-2LSAAC: a two-level model for discrimination of membrane proteins and their types by extending the notion of SAAC into Chou's pseudo amino acid composition. *Journal of Theoretical Biology,* **442**, 11–21 [PubMed:29337263] [doi:10.1016/j.jtbi.2018.01.008].
16. Butt, A. H., Khan, S. A., Jamil, H., Rasool, N., and Khan, Y. D. (2016) A prediction model for membrane proteins using moments based features. *BioMed research international,* **2016** [PubMed:26966690] [PubMed Central:PMC4761391] [doi:10.1155/2016/8370132].
17. Zou, Q., Li, X., Jiang, Y., Zhao, Y., and Wang, G. (2013) BinMemPredict: a web server and software for predicting membrane protein types. *Current Proteomics,* **10**(1), 2–9.

18. Cedano, J., Aloy, P., Pérez-Pons, J. A., and Querol, E. (1997) Relation between amino acid composition and cellular location of proteins. *Journal of Molecular Biology,* **266**(3), 594–600 [PubMed:9067612] [doi:10.1006/jmbi.1996.0804].
19. Alballa, M. and Butler, G. (2020) Integrative approach for detecting membrane proteins. *BMC bioinformatics,* **21**, 1–25 [PubMed:33349234] [PubMed Central:PMC7751106] [doi:10.1186/s12859-020-03891-x].
20. Ali, F. and Hayat, M. (2015) Classification of membrane protein types using Voting Feature Interval in combination with Chou's Pseudo Amino Acid Composition. *Journal of theoretical biology,* **384**, 78–83 [PubMed:26297889] [doi:10.1016/j.jtbi.2015.07.034].
21. Han, G.-S., Yu, Z.-G., and Anh, V. (2014) A two-stage SVM method to predict membrane protein types by incorporating amino acid classifications and physicochemical properties into a general form of Chou's PseAAC. *Journal of Theoretical Biology,* **344**, 31–39 [PubMed:24316387] [doi:10.1016/j.jtbi.2013.11.017].
